# RSM and GA-BP assisted optimization of enzyme-ultrasound assisted extraction for mulberry fruit polysaccharides and comparison of their protective effects on NAFLD with hot water extraction polysaccharide

**DOI:** 10.3389/fnut.2026.1915643

**Published:** 2026-07-30

**Authors:** Hengpu Zhou, Zhangyun Wu, Mingting Jiang, Liang Cheng, Meiqiu Yan, Xinglishang He, Guiyuan Lv, Suhong Chen

**Affiliations:** School of Pharmaceutical Sciences, Zhejiang Chinese Medical University, Hangzhou, Zhejiang, China

**Keywords:** enzyme-ultrasound assisted extraction, gut microbiota, mulberry fruit polysaccharides, non-alcoholic fatty liver disease, RSM

## Abstract

**Background:**

Non-alcoholic fatty liver disease (NAFLD) is triggered by gut-liver axis disorders; enzyme-ultrasound assisted extraction (EAUE) better retains bioactive mulberry polysaccharides (MP) than hot water extraction. This work optimized EAUE by RSM and GA-BP to compare the gut-hepatic protective capacity of two MP preparations and reveal their microecological regulatory mechanism.

**Methods:**

We optimized EAUE conditions and characterized the structural features of EAUE-derived MP (MPEU). A 30-day concurrent modeling and MP-administration mouse trial was conducted with biochemical detection and 16S rRNA sequencing to assess MPEU and hot water extraction MP (MPHW) *in vivo* activity.

**Results:**

RSM yielded predictions closer to experimental values, and its optimal extraction conditions were determined as extraction time 107 min, liquid-to-solid ratio 33:1, enzyme addition amount 350 mg, and extraction temperature 52 °C, yielding MP at 10.06 ± 0.03%. MPEU retained complete glycosidic structures and outperformed MPHW in mitigating hepatic steatosis, intestinal damage and inflammation, by restoring SCFA-producing commensal bacteria and suppressing pro-inflammatory flora.

**Conclusion:**

In this study, RSM is preferable for EAUE optimization. Structurally intact MPEU exerts stronger NAFLD relief via remodeling gut microecology, promising as nutritional functional food ingredients.

## Introduction

Non-Alcoholic Fatty Liver Disease (NAFLD) is the most prevalent chronic liver disorder globally, characterized by excessive lipid accumulation within hepatocytes accompanied by oxidative stress, chronic low-grade inflammation, and gut-liver axis dysfunction (1). Without timely intervention, it may progress to non-alcoholic steatohepatitis, cirrhosis (2, 3), or even hepatocellular carcinoma (4). The pathogenesis of NAFLD is closely linked to energy excess and ectopic lipid deposition (5). Key drivers of disease progression include gut microbiota dysbiosis, intestinal mucosal barrier damage, and endotoxemia induced by long-term high-sugar, high-fat diets. High-fat diets disrupt gut microbiota homeostasis, reducing the abundance of beneficial bacteria producing short-chain fatty acids (SCFAs) while promoting the proliferation of pro-inflammatory bacteria (6, 7). This damages intestinal tight junctions, allowing toxic metabolites like lipopolysaccharide (LPS) to enter the bloodstream. Currently, no specific drugs for NAFLD exist clinically. Bioactive compounds derived from natural foods, however, have gained attention in NAFLD prevention and treatment research due to their high safety profile and multi-target regulatory advantages (8, 9).

Mulberry fruit, a widely cultivated fruit with both medicinal and edible uses, is rich in active components such as polysaccharides, flavonoids, and anthocyanins (10–15). Among these, mulberry fruit polysaccharides (MP), as water-soluble heteropolysaccharides, have been demonstrated to possess antioxidant, anti-inflammatory, and glucose-lipid metabolism-regulating bioactivities (16–18). Ai employed an enzyme-assisted extraction (EAE) method using pectinase and pectin lyas, achieving yields of 7.7 and 8.3%, respectively (19). Li utilized a high-shear-assisted hot water extraction method, yielding 9.95% (20). Recent studies indicate that MP can alleviate metabolic diseases by reshaping gut microbiota structure, increasing SCFA production, and repairing the intestinal mucosal barrier (21). However, systematic research on MP’s role in improving NAFLD and its mechanism of exerting hepatoprotective effects through gut microbiota regulation remains scarce. Its potential as a functional ingredient in NAFLD prevention and treatment warrants further exploration.

The extraction efficiency and quality of MP directly determine its biological activity and application value. Traditional hot water extraction suffers from low yield, time-consuming processes, high energy consumption, and susceptibility to polysaccharide structural degradation (22, 23). Enzyme-Assisted Ultrasound Extraction (EAUE) is a highly efficient, eco-friendly extraction technique. It employs enzymatic digestion to disrupt mulberry fruit cell walls and utilizes ultrasound to accelerate mass transfer, significantly enhancing polysaccharide extraction yield and activity (24, 25). Response Surface Methodology (RSM) efficiently optimizes extraction processes involving multiple interacting factors, providing scientific basis for MP’s large-scale production (26–28). Meanwhile, Genetic Algorithm-optimized Back Propagation (GA-BP) neural networks, with their strong ability to model high-order nonlinear relationships and avoid local optima, offer a complementary approach to RSM for refining extraction parameter prediction and global optimization (29, 30). To date, no studies have reported the use of RSM and GA-BP to optimize the EAUE process for MP or investigated its potential to improve NAFLD through gut microbiota regulation.

Extraction techniques directly determine the molecular integrity of plant polysaccharides. Conventional hot-water extraction causes severe degradation of glycosidic bonds and long sugar chains under prolonged high temperature, while enzyme-ultrasound assisted extraction preserves intact active structures under mild conditions. Previous studies have validated that complete polysaccharide skeletons are critical for regulating gut microbiota, repairing intestinal barriers and restoring the gut-liver axis; structural damage greatly weakens the anti-NAFLD bioactivity of polysaccharides (31–33).

This study optimized the enzyme-assisted ultrasonic extraction of mulberry polysaccharides via RSM and GA-BP to acquire high-activity MP. Using a high-fat-high-sugar NAFLD mouse model, we compared the hepatoprotective effects of MP prepared via EAUE (MPEU) and hot water extraction (MPHW) by evaluating hepatic lipid accumulation, oxidative stress and inflammatory responses, and further clarified how MPEU alleviates NAFLD via regulating gut microbiota and restoring gut-liver axis homeostasis. This work offers theoretical guidance and experimental data for the industrial production of mulberry polysaccharides and their application as functional food ingredients for NAFLD nutritional intervention.

## Materials and methods

### Materials

Experimental Reagents Cellulase (50 u/mg) and glucose (purity ≥98%) (Batch Nos.: KR383969, KR390636) were purchased from Shanghai Yuanye Biotechnology Co., Ltd. (Shanghai, China). Mouse superoxide dismutase (SOD) and glutathione peroxidase (GSH-Px) (Catalog Nos.: RXW201865M, RX2DW222266) assay kits were purchased from Quanzhou Ruixin Biotechnology Co., Ltd. (Jiangsu, China). TCH (A111-1-1), TG (A110-1-1), and ALT+AST (Z002-1-1) kits were purchased from Nanjing Jiancheng Biological Engineering Institute (Jiangsu, China). Lipopolysaccharide (LPS, JL52811) and Free Fatty Acid (FFA, JLT-1313) kits were purchased from Jianglai Biological (Jiangsu, China). Occludin, Claudin-1 and ZO-1 (Cat No. 27260-1-AP, 28674-1-AP and 21,773-1-AP) polyclonal antibody were purchased from Wuhan Sanying (Wuhan, China). Ethanol and methanol were of chromatographic grade. Water was ultrapure water. Reagents included analytical-grade sulfuric acid and phenol Ltd. (Batch Nos.: 10021661, S328111100G) purchased from Sinopharm Chemical Reagent Co., (Shanghai, China).

### Extraction process and characterization of MPEU

#### MP extraction process

Mulberry fruits were pulverized through a 40-mesh sieve. Ten grams of mulberry powder were weighed and extracted using different methods with water. After extraction, the mixture was vacuum filtered, and the filtrate was collected and concentrated under reduced pressure at 50 °C to 50 mL. While stirring, 200 mL of anhydrous ethanol was added. The mixture was incubated at 4 °C overnight, then centrifuged at 3500 × g for 10 min. The precipitate was collected, freeze-dried for 12 h, yielding MP. Polysaccharide content was determined using the phenol-sulfuric acid method as follows:

Precisely weigh 1 mg of MP into a 10 mL volumetric flask, dissolve with water to prepare a 0.1 mg/mL test solution. Transfer different volumes of test and reference solutions, dilute with water to 1 mL, add 0.5 mL of 5% phenol solution, then add 2.5 mL of sulfuric acid. Boil in a water bath for 15 min, cool in an ice bath for 5 min. Pipette 200 μL of each sample into a 96-well plate and measure the absorbance at 489 nm.

#### Extraction process study

Hot Water Extraction: Weigh 10 g mulberry powder, add 200 mL pure water, reflux extract for 60 min, filter, concentrate under reduced pressure, and precipitate with ethanol to obtain mulberry polysaccharides.

Ultrasonic extraction: Weigh 10 g mulberry powder, add 200 mL pure water, sonicate at 50 °C (500 W, 20 Hz) for 60 min, filter, concentrate under reduced pressure, and precipitate with alcohol to obtain mulberry polysaccharides.

Enzymatic extraction: Weigh 10 g mulberry powder, add 200 mL pure water and 300 mg cellulase, adjust pH to 6, shake at 50 °C (200 rpm) for 60 min extraction, inactivate in boiling water bath for 10 min, filter, concentrate under reduced pressure, and precipitate with alcohol to obtain mulberry polysaccharides.

Ultrasound-assisted enzymatic extraction: Weigh 10 g mulberry powder, add 200 mL pure water and 200 mg cellulase, adjust pH to 6, extract by ultrasonication (500 W, 20 Hz) at 50 °C for 60 min, inactivate by boiling water bath for 10 min, filter, concentrate under reduced pressure, and precipitate with alcohol to obtain mulberry polysaccharides.

#### Single-factor investigation

Using polysaccharide yield as the indicator, the following factors were investigated: extraction time (15, 30, 60, 90, 120 min), liquid-to-solid ratio (10, 20, 30, 34, 35), enzyme dosage (100, 200, 300, 400, 500 mg), extraction temperature (30, 40, 50, 60, 70 °C), and ultrasonic power (500, 450, 400, 350, 200 W) on polysaccharide extraction.

#### RSM extraction process optimization

Building upon single-factor experiments, four factors - extraction time, liquid-to-solid ratio, enzyme addition amount and extraction temperature - were selected. Using polysaccharide yield as the response metric, a Box–Behnken response surface design was employed to investigate their effects. Factors and levels as detailed in Table 1.

**Table 1:** Factors and levels for response surface experiments.

Level	Factor			
	Extraction Time (min)	Liquid-to-solid Ratio	Enzyme addition Amount (mg)	Extraction Temperature (°C)
–1	30	20	300	40
0	60	30	400	50
1	90	40	500	60

#### GA-BP extraction process optimization

Based on the method in the reference (36), 29 sets of RSM experimental data were normalized for independent variables and response values in Matlab, then randomly divided into training and testing sets at a 7:3 ratio; A BP neural network was constructed. The BP neural network prediction values served as the fitness function, and the core parameters of the genetic algorithm were set to perform global optimization of the independent variables. The normalized parameters obtained through optimization were denormalized to restore the actual process parameters and predict the corresponding extraction rates, thereby obtaining the optimal process conditions for the GA-BP method.

#### FT-IR

Based on the method in the reference (37, 38), take 2 mg of dried MP sample and thoroughly grind it with KBr powder (200 mg) to form a transparent thin plate; Scan using a Nicolet IS50 Fourier Transform Infrared Spectrometer (Thermo Fisher Scientific, Waltham, USA) in the wavenumber range of 400–4,000 cm^−1^ at a resolution of 4 cm^−1^, with 32 scans. Record the infrared spectrum and analyze characteristic polysaccharide functional groups.

#### Monosaccharide composition

Take 10 mg of MP sample, add 5 mL of 2 mol/L trifluoroacetic acid solution, and hydrolyze at 110 °C for 6 h. Evaporate the hydrolysate to dryness under reduced pressure, wash repeatedly with methanol to remove acid, evaporate to dryness, and dissolve in ultrapure water. After derivatization on a PMP column, the solution was filtered through a 0.22 μm membrane and analyzed by HPLC (Agilent Technologies, CA, USA). Using monosaccharide standards (glucose, mannose, galactose, arabinose, xylose) as references, the composition and molar ratios of each monosaccharide in MP were quantified by external standard method.

Chromatographic conditions: Agilent 1,200 liquid chromatograph (Agilent Technologies, Beijing, China), Agilent XB-C18 column (4 × 250 mm), acetonitrile (A)-0.1 mol/L ammonium acetate (B), mobile phase gradient: 0–18 min, 16.8-12%A; 18–18.2 min, 12%A; 18.2–22 min, 12-22%A; 22–23 min, 22%A; 23–25 min, 22-20%A; 25–26 min, 20%A; 26–42 min, 20-30%A; 42–60 min, 30%A. Flow rate 1.0 mL/min, 30 °C, injection volume 5 μL, 250 nm.

#### Congo red

Prepare a 0.1 mmol/L Congo Red solution. Mix equal volumes with MP sample solutions of varying concentrations, with distilled water serving as a blank control. After standing at room temperature for 10 min, scan using a UV–visible spectrophotometer (Shimadzu, Kyoto, Japan) to scan the wavelength range of 400–600 nm and determine the maximum absorption wavelength, analyzing whether the polysaccharide exhibits a triple-helix structure.

#### Electron microscopy

Take an appropriate amount of dried MP sample, dissolve in ultrapure water to prepare a 0.5 mg/mL polysaccharide solution, dispense 5 μL onto a clean conductive sample stage, and allow to air-dry at room temperature. Perform gold coating on the sample surface using an ion sputter coater (McAudi, Fujian, China) (coating thickness: 10–20 nm). Observe the microscopic morphology of the polysaccharides under a scanning electron microscope at different magnifications and capture images.

### Animal experiment design

#### Animals

SPF-grade male ICR mice (4–6 weeks old, body weight 30 ± 2 g) were purchased from Hangzhou Qizhen Laboratory Animal Technology Co., Ltd. (Experimental Animal License No.: SCXK(Zhe)2022–0005). Animal experiments were approved by the Zhejiang University of Chinese Medicine Laboratory Animal Management and Ethics Committee (Approval No.: 20250707-02). Prior to the experiment, all animals underwent a 3-day acclimatization period. They were housed in a constant environment with temperature (20 ± 2 °C), humidity (55 ± 5%), and a 12-h light/dark cycle. Animals had free access to food and water throughout the experiment.

#### Animal grouping and administration

After 3 days of acclimation feeding, animals were randomly divided into 4 groups (*n* = 6) based on body weight using SPSS 26.0: Normal Control Group (NG), Model Control Group (MG), MPEU (200 mg/kg), and MPHW (200 mg/kg). A high-sugar high-fat diet (It was composed of 72.5% standard chow diet, 10% sucrose, 10% lard, 5% egg yolk powder, 2% cholesterol, and 0.5% bile salt) was used to replicate the non-alcoholic fatty liver mouse model. During the modeling period, the corresponding drugs were administered to each treatment group by gavage, while the NG group and MG model group were gavaged with distilled water. The gavage volume was 0.1 mL/10 g body weight, and the administration was performed once a day for 30 consecutive days.

Following the final administration, mice were intraperitoneally anesthetized with sodium pentobarbital (50 mg/kg body weight). Blood and tissues were collected under anesthesia for biochemical detection and histopathological examination. After sampling, all mice were humanely euthanized by gradual CO₂ inhalation at a flow rate displacing 20% of the chamber volume per minute, in strict accordance with our institutional animal ethics guidelines.

#### Body weight

During the dosing period, mouse body weight was measured every 10 days.

#### Grip strength

Place the mouse on the grip strength tester, maintaining the torso in a horizontal position, and allow only the front paws to contact the grid before any measurements. Gently pull back the mouse’s tail to ensure it grasps the top of the grid while keeping the torso horizontal, and record the grip strength value displayed on the screen.

#### Spontaneous activity

Place the mouse in the spontaneous activity monitor in a quiet environment. After a 5-min adaptation period, record the number of activities performed by the mouse within the next 5 min.

#### Serum assay kit detection

Collect mouse blood into centrifuge tubes. Incubate blood at 37 °C water bath for 30 min, then centrifuge at 4000 × g for 10 min. Collect the supernatant as serum. Detect serum levels of SOD, GSH, TC, TG, ALT, AST, LPS and FFA according to the kit instructions.

#### Liver assay kit detection

Collect 0.1 g of mouse liver tissue and add 0.9 mL PBS. Grind using a low-temperature tissue grinder for 1 min, then centrifuge at 10,000 × g for 10 min. The supernatant was collected for the detection of hepatic homogenate indicators. Detect SOD, GSH, IL-6, and TNF-*α* levels in the liver homogenate according to the kit instructions.

#### HE staining

Take paraffin sections of liver and colon tissue. After dewaxing and rehydration, perform hematoxylin staining for 5 min, acid-alcohol differentiation for 30 s, and eosin staining for 2 min. Dehydrate with graded ethanol, clear with xylene, mount with neutral binder, and observe liver histopathology under a light microscope.

#### Oil Red O staining

Fresh liver tissue was OCT-embedded and sectioned into 8 μm frozen sections, air-dried at room temperature for 30 min. Rinse with 60% isopropanol for 1 min, stain with Oil Red O solution for 15 min, and differentiate with 60% isopropanol until background is colorless. Counterstain nuclei with hematoxylin for 5 min, rinse under running water to restore blue color, mount with glycerol-gelatin, and observe hepatic lipid droplet distribution under microscopy.

#### Immunohistochemistry

Take 4 μm paraffin sections of colon tissue, deparaffinize to water, then block endogenous peroxid with 3% H₂O_2_ at room temperature for 10 min to block endogenous peroxidase activity; antigen retrieval was performed using citrate buffer (pH 6.0) at high temperature and pressure for 15 min, followed by three rinses with pre-chilled PBS; blocked with 5% bovine serum albumin (BSA) at room temperature for 30 min; primary antibody was added and incubated overnight at 4 °C; after PBS washing, secondary antibody was added and incubated at room temperature for 1 h; Develop with DAB color developer for 3–5 min, counterstain nuclei with hematoxylin, dehydrate, clear, and mount slides. Observe under microscope and quantify positive expression area and optical density values using ImageJ software.

#### Determination of SCFA

Collect fresh mouse fecal samples (approximately 100 mg), rapidly freeze in liquid nitrogen, and store at −80 °C. Add sterile PBS to the sample, vortex to mix, and sonicate for 30 min. Centrifuge at 10,000 × g at 4 °C for 15 min, then collect the supernatant. Acidify with 25% metaphosphoric acid solution (4:1 v/v), incubate at room temperature for 30 min, centrifuge again, and filter the supernatant through a 0.22 μm organic membrane. Detect using gas chromatography with HP-FFAP capillary column separation and hydrogen flame ionization detector. Quantify SCFAs concentrations using external standard method (acetic acid, propionic acid, butyric acid, isobutyric acid, valeric acid, isovaleric acid standards).

#### Gut microbiota

Collect fresh mouse feces (NG, MG and MPEU), rapidly freeze in liquid nitrogen, and store at −80 °C. Extract total DNA from samples using a fecal genomic DNA extraction kit according to the manufacturer’s instructions. Detect DNA purity and concentration via 1% agarose gel electrophoresis. Targeted PCR amplification was performed using specific primers for the V3-V4 region of the bacterial 16S rRNA gene. Amplified products were recovered from agarose gel, quantified, and used to construct sequencing libraries. High-throughput sequencing was conducted on the Illumina MiSeq platform. Gut microbiota sequencing was performed by Majorbio (Shanghai, China).

### Statistical analysis

Data were processed and analyzed using SPSS 19.0 and GraphPad 9.0. Results are expressed as mean ± standard deviation (X ± SD). Pairwise comparisons were performed using t-tests, while multiple group comparisons employed one-way analysis of variance (ANOVA). *p* < 0.05 was considered statistically significant.

## Results

### Comparison of extraction methods

The traditional polysaccharide extraction method is hot water extraction. Results showed that the polysaccharide content obtained by hot water extraction (7.06 ± 0.30%) was higher than that obtained by ultrasonic extraction alone (6.76 ± 0.16%) and enzymatic extraction (4.82 ± 0.50%), but lower than that obtained by ultrasonic-assisted enzymatic extraction (7.87 ± 0.10%). Therefore, subsequent experiments selected ultrasonic-assisted enzymatic extraction, with single-factor and response surface optimization applied to its process.

### Single-factor experiment results

Extraction time and temperature yielded similar results: within the selected range, polysaccharide extraction rates initially increased then declined. This may stem from excessive ultrasound duration or high temperatures damaging polysaccharide structures, inactivating co-enzymes, and intensifying side reactions, leading to reduced polysaccharide content. Consequently, extraction at 90 min and 50 °C was selected as the intermediate level for subsequent experiments.

Similar results were observed for the liquid-to-solid ratio and enzyme addition amount, showing an initial increase followed by stabilization. This indicates that polysaccharides can be extracted relatively completely at a 20:1 liquid-to-solid ratio and an enzyme addition amount of 400 mg. Therefore, a liquid-to-solid ratio of 20:1 and an enzyme addition amount of 400 mg were selected for subsequent experiments.

**Figure 1:**
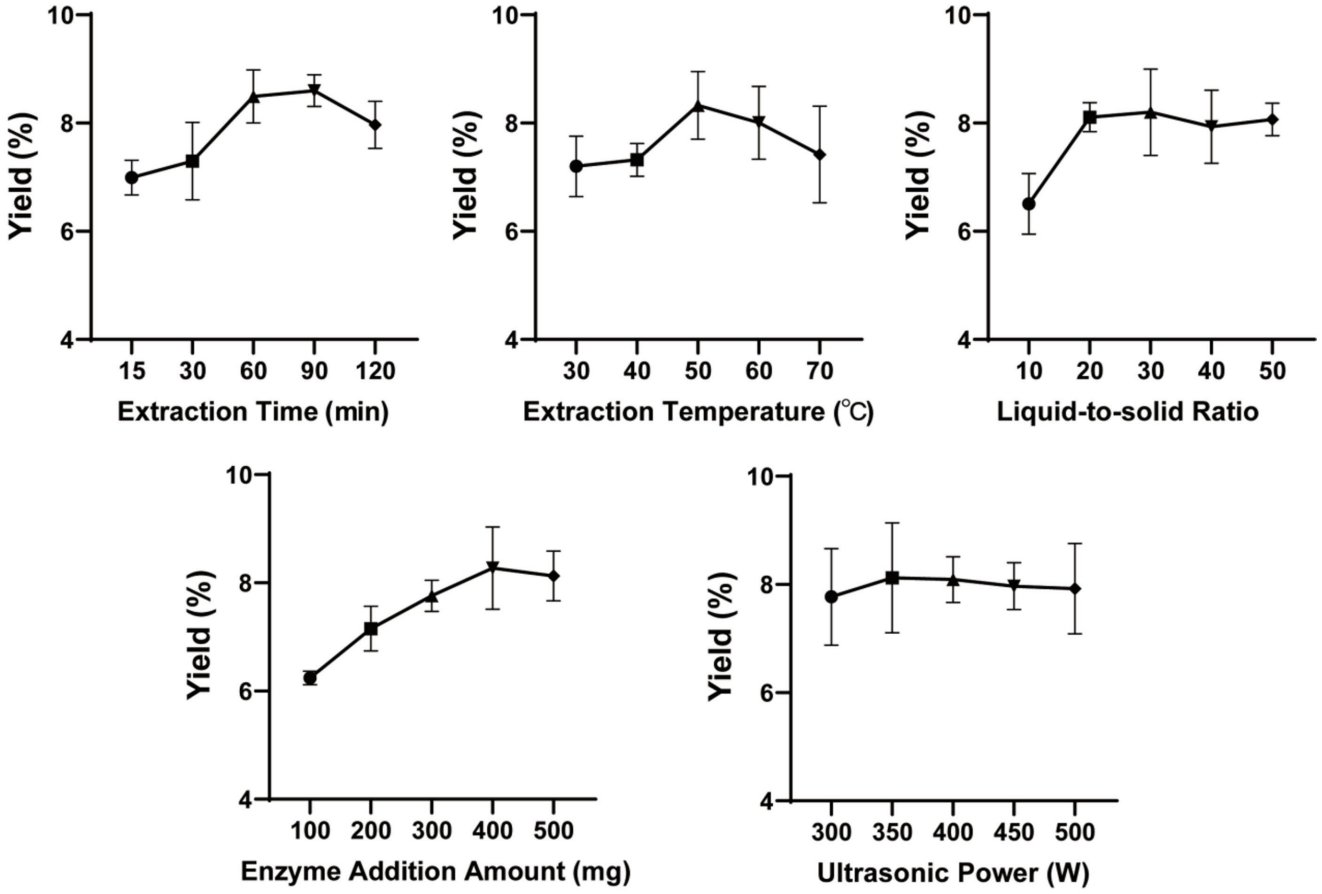
Single-factor results.

Ultrasonic power results showed minimal impact on polysaccharide yield within the 300–500 W range. Consequently, the machine’s default power setting (500 W) was adopted for subsequent experiments (Figure 1).

### RSM

Based on the RSM results, a multiple regression model was fitted to establish the relationship between polysaccharide yield (Yield) and extraction time (A), liquid-to-solid ratio (B), enzyme dosage (C), and extraction temperature (D) is as follows: Yield = −43.45116 + 0.275173 A + 0.578797 B + 0.091021 C + 0.606143 D + 0.000520 A B − 0.000080 A C − 0.000782 A D − 0.000529 B C + 0.003121 B D − 0.000359\u00B0C D − 0.001266 A^2^–0.009351 B^2^–0.000066 C^2^–0.004773 D^2^.

**Figure 2:**
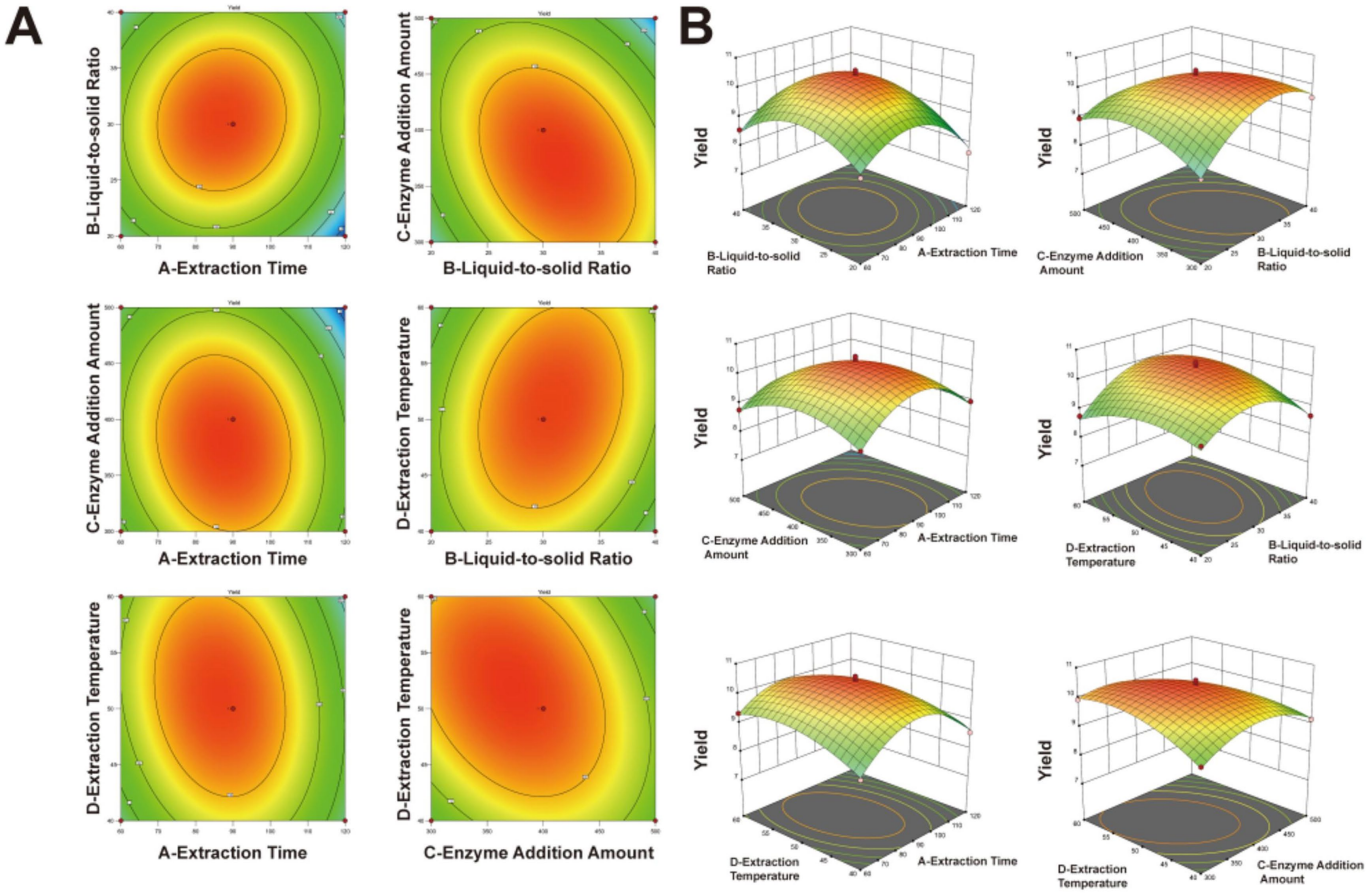
Interaction plots of various factors. **(A)** Contour plot. **(B)** 3D response surface plot.

The model *p*-value < 0.0001 indicates that this regression model is highly significant and can explain the vast majority of variation in polysaccharide yield observed in the experiment. The *p*-value for the lack of fit is 0.7405 (> 0.05), indicating that the lack of fit is not significant. This suggests that the model fits the experimental data well, with no significant unexplained variation. Among the linear terms, extraction time (A) and enzyme addition amount (C) exerted extremely significant effects on polysaccharide yield (*p* < 0.0001); while liquid-to-solid ratio (B) and extraction temperature (D) showed significant effects (*p* < 0.05). Among the interaction terms, AC, AD, BC, BD, and CD all exhibited significant or extremely significant effects (*p* < 0.05 or 0.0001), indicating pronounced synergistic or antagonistic interactions among the factors. Among the quadratic terms, A^2^, B^2^, C^2^ and D^2^ all exhibited extremely significant effects (*p* < 0.0001), further validating the significant nonlinear relationship between factors and polysaccharide yield and confirming the appropriateness of using a quadratic polynomial model for fitting (Tables 2, 3 and Figure 2).

**Table 2:** Response surface experiment results of MP extraction process.

STD	RUN	A-extraction Time	B-Liquid-to-solid ratio	C-Enzyme addition amount	D-extraction Temperature	Yield (%)
27	1	90	30	400	50	10.54
23	2	90	20	400	60	8.75
24	3	90	40	400	60	9.43
10	4	120	30	400	40	8.56
25	5	90	30	400	50	10.36
17	6	60	30	300	50	8.88
8	7	90	30	500	60	8.53
6	8	90	30	500	40	9.16
20	9	120	30	500	50	7.89
22	10	90	40	400	40	8.65
3	11	60	40	400	50	8.56
15	12	90	20	500	50	8.95
18	13	120	30	300	50	8.95
4	14	120	40	400	50	8.35
11	15	60	30	400	60	9.34
21	16	90	20	400	40	9.21
1	17	60	20	400	50	8.47
29	18	90	30	400	50	10.33
5	19	90	30	300	40	9.12
16	20	90	40	500	50	7.97
19	21	60	30	500	50	8.77
7	22	90	30	300	60	9.93
12	23	120	30	400	60	8.36
9	24	60	30	400	40	8.60
2	25	120	20	400	50	7.63
28	26	90	30	400	50	10.12
14	27	90	40	300	50	9.57
26	28	90	30	400	50	10.46
13	29	90	20	300	50	8.44

**Table 3:** Analysis of variance for response surface methodology.

Source	Sum of squares	df	Mean square	*F*-value	*P*-value	Significance
Model	17.27	14	1.23	68.3	<0.0001	***
A-Extraction Time	0.6863	1	0.6863	38	<0.0001	***
B-Liquid-to-solid Ratio	0.0965	1	0.0965	5.34	0.0366	*
C-Enzyme Addition Amount	1.09	1	1.09	60.24	<0.0001	***
D-Extraction Temperature	0.0895	1	0.0895	4.96	0.043	*
AB	0.0975	1	0.0975	5.4	0.0357	*
AC	0.2279	1	0.2279	12.62	0.0032	**
AD	0.2202	1	0.2202	12.19	0.0036	**
BC	1.12	1	1.12	62.01	<0.0001	***
BD	0.3896	1	0.3896	21.57	0.0004	***
CD	0.5144	1	0.5144	28.48	0.0001	***
A^2^	8.42	1	8.42	466.04	<0.0001	***
B^2^	5.67	1	5.67	314.04	<0.0001	***
C^2^	2.85	1	2.85	158.02	<0.0001	***
D^2^	1.48	1	1.48	81.82	<0.0001	***
Residual	0.2529	14	0.0181			
Lack of fit	0.1558	10	0.0156	0.6425	0.7405	Not significant
Pure error	0.097	4	0.0243			
Cor total	17.52	28				

*p < 0.05, **p < 0.01, ***p < 0.001.

### GA-BP

**Figure 3:**
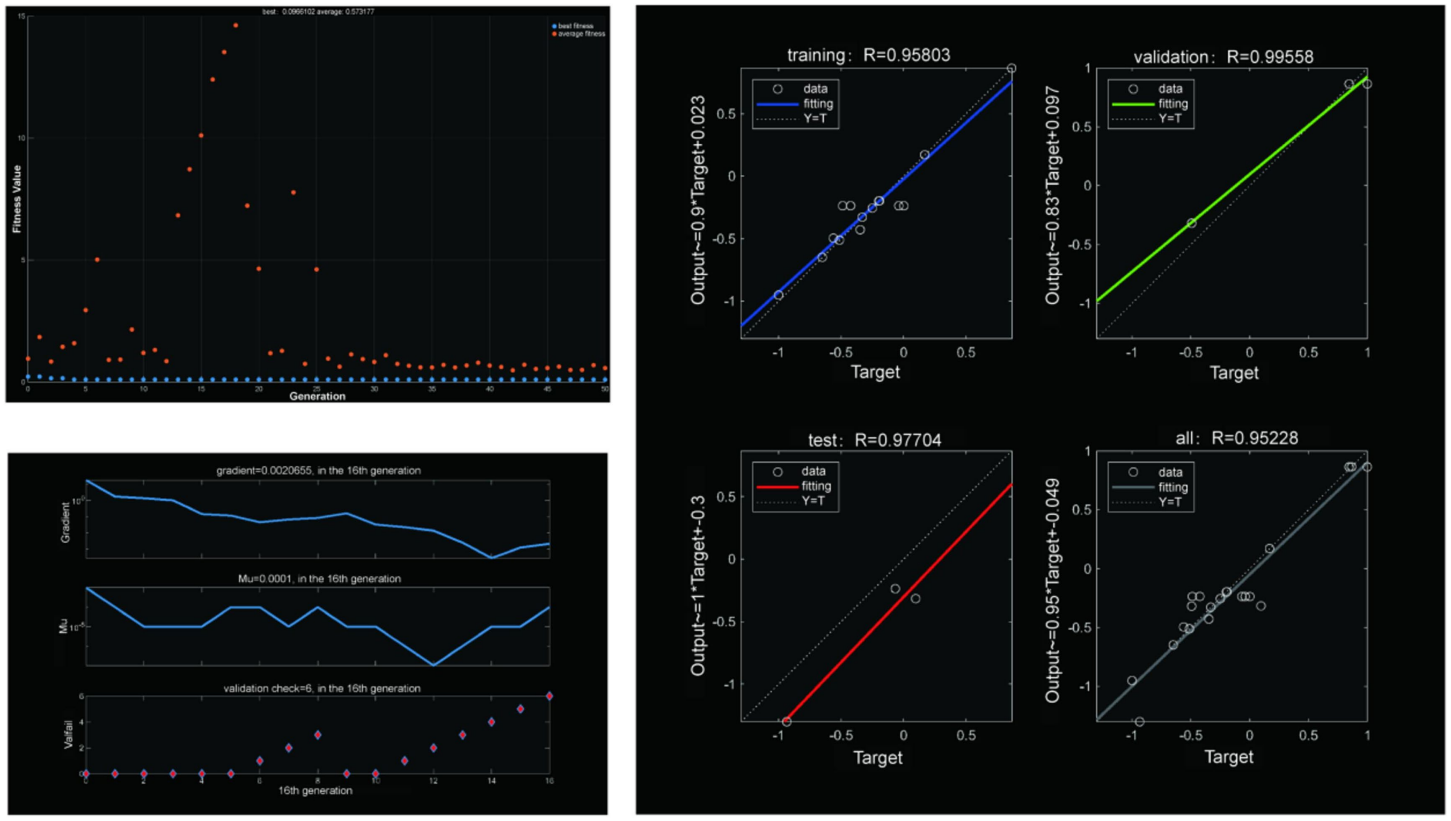
GA-BP result plot.

This study constructed a GA-BP neural network model for predicting polysaccharide extraction rates. Four enzymatic hydrolysis-related process parameters served as inputs (input layer with 4 nodes), while polysaccharide extraction rate was the output (output layer with 1 node). The optimal network structure was determined by comparing the mean squared error (MSE) of training sets across different hidden layer node counts: When the hidden layer had 6 nodes, the MSE decreased to its minimum value of 0.038296, thus confirming the network structure as 4-6-1; Genetic algorithms were employed to optimize the weights and thresholds of this BP network, yielding the optimized weight matrices W1 (6 × 4) and W2 (1 × 6), along with threshold matrices B1 (6 × 1) and B2 (1 × 1). The genetic algorithm achieved fitness convergence within 50 generations, with the optimal fitness stabilizing at 0.0966102. Performance comparisons demonstrate that the GA-BP model achieves significantly higher prediction accuracy than the traditional BP network: The GA-BP model achieved lower Mean Absolute Error (MAE), Mean Squared Error (MSE), Root Mean Squared Error (RMSE), and Mean Absolute Percentage Error (MAPE) of 0.31088, 0.14395, 0.37941, and 3.3629%, respectively (with some metrics further optimized to 0.14941, 0.049266, 0.22196, 1.6896%), while the traditional BP network recorded maximum values of 1.0985, 2.0655, 1.4372, and 12.8206% for corresponding metrics. Verification on 8 test samples showed GA-BP’s predicted values exhibited smaller deviations from actual values (e.g., sample error only −0.0154). The model achieved fitting correlation coefficients >0.95 across the training set (*R* = 0.95803), validation set (*R* = 0.99558), and test set (*R* = 0.97704), demonstrating strong generalization capability (Figure 3).

### Validation of optimal extraction process

The RSM method identified the optimal extraction process as follows: extraction time: 107.59 min, liquid-to-solid ratio: 32.78, enzyme addition amoun: 349.58 mg, extraction temperature: 52.26 °C, with a predicted yield of 9.96%. After appropriate adjustments and validation of the extraction conditions (extraction time: 107 min; liquid-to-solid ratio: 33:1; enzyme addition amount: 350 mg; extraction temperature: 52 °C), the final polysaccharide yield reached 10.06 ± 0.03%, closely matching the predicted value.

The GA-BP method identified the optimal extraction process as follows: extraction time 115 min, liquid-to-solid ratio 40:1, enzyme addition amount 320 mg, extraction temperature 56 °C, with a predicted yield of 12.23%. Validation using these conditions yielded a final polysaccharide yield of 10.04 ± 0.09%, showing a significant deviation from the predicted value.

In this study, the relative deviation between the polysaccharide extraction rate predicted by the RSM and the experimentally verified value was < 5%, indicating an extremely high degree of fit. This stems primarily from RSM’s reliance on quadratic polynomial regression modeling, which better accommodates the moderate-to-low nonlinear interactions among process factors in this study. Furthermore, its optimization process leverages the densely sampled regions of the Box–Behnken design, enabling more precise local fitting to the actual process. In contrast, the GA-BP neural network prediction exhibits a relative deviation exceeding 5% from actual values, indicating significantly larger errors. The core reason lies in GA-BP modeling relying solely on 29 experimental data sets - a small sample size - with a lack of high-extraction-rate samples in the training set. Consequently, the model can only predict high values beyond the data range through extrapolation. Additionally, the model exhibits mild overfitting. Given the low nonlinear intensity of the process studied, GA-BP’s advantage in complex nonlinear mapping could not be leveraged. Instead, overlearning local features led to inflated predictions. This outcome confirms that process optimization method selection must align with system complexity. RSM demonstrates greater practical applicability in this study, while GA-BP requires enhancing prediction reliability through measures such as supplementing high-value region samples and optimizing model structure.

### MP characterization

**Figure 4:**
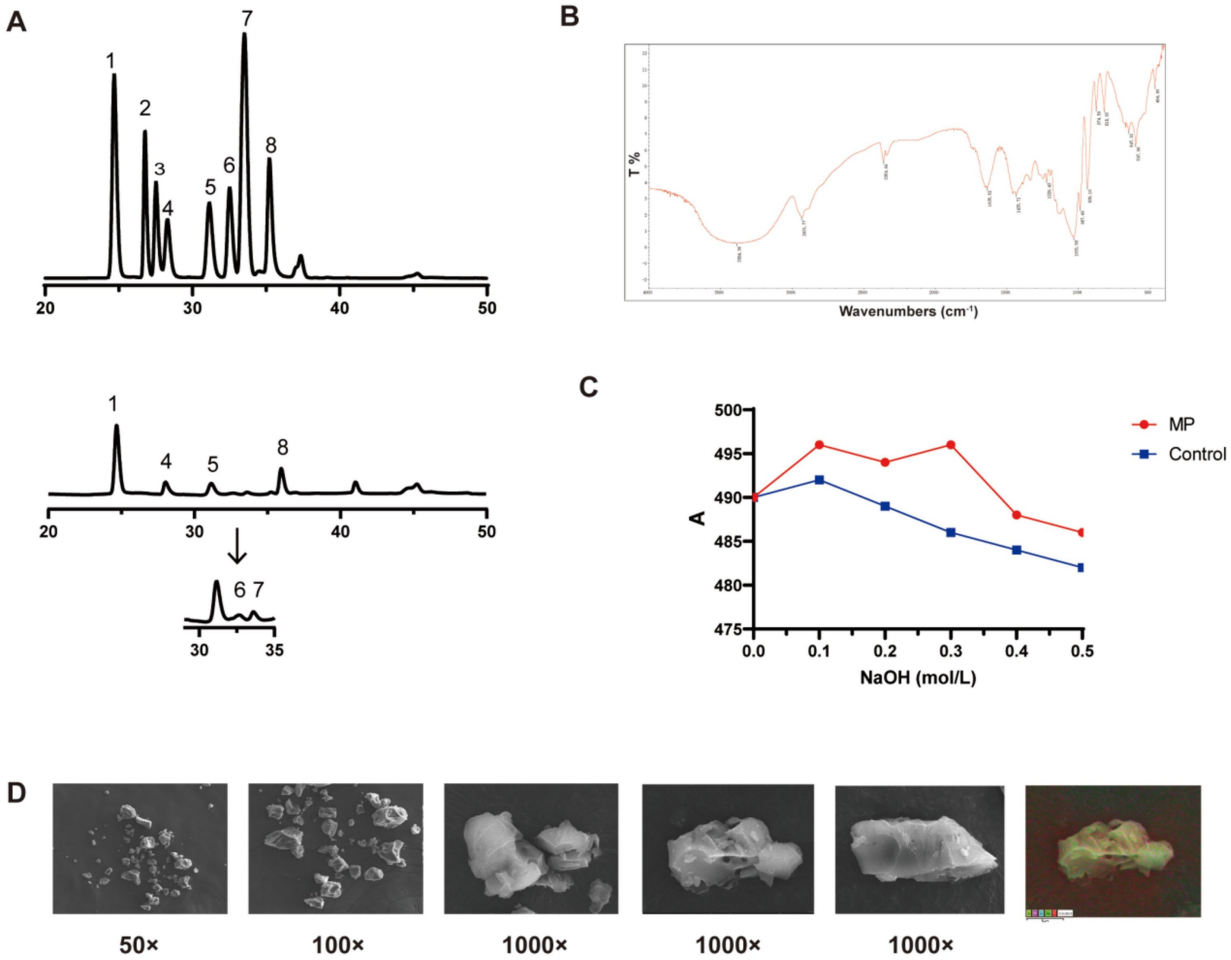
Characterization plots of MP. **(A)** Monosaccharide composition. **(B)** FT-IR spectrum. **(C)** Congo red test. **(D)** Electron micrograph. 1. Mannose, 2. Rhamnose, 3. Glucuronic acid, 4. Galacturonic acid, 5. Glucose, 6. Galactose, 7. Arabinose, 8. Fucose.

Detected monosaccharides in the sample include mannose, galacturonic acid, glucose, galactose, arabinose, and fucose (Figure 4A). Their relative molar percentages are 48.64: 19.90: 13.78: 1.10: 0.69: 15.89.

As depicted in Figure 4B, the broad valley at 3384.36 cm^−1^ corresponds to hydroxyl O-H stretching, and 2931.77 cm^−1^ belongs to sugar backbone C-H stretching. The strong valley at 1635.52 cm^−1^ originates from bound water and galacturonic acid C=O vibration. The characteristic pyranose glycosidic fingerprint valley appears at 1031.58 cm^−1^, with weak auxiliary signals at 874.59 and 818.93 cm^−1^. Although galacturonic acid was detected in MPEU, no obvious valley of esterified carboxyl at 1750 cm^−1^ was observed, reflecting low polysaccharide methylation.

Congo red staining results showed that MP did not exhibit characteristic red shift upon binding with Congo red dye under alkaline conditions, indicating a non-helical polysaccharide structure lacking the typical triple-helix conformation (Figure 4C).

SEM revealed that mulberry polysaccharides exist as irregular granular aggregates under low magnification (50×, 100×). At high magnification (1000×), they appear as smooth-surfaced amorphous masses or flakes without distinct crystalline features, reflecting typical solid-state aggregation characteristics (Figure 4D).

### MP improves overall condition in NAFLD mice

**Figure 5:**
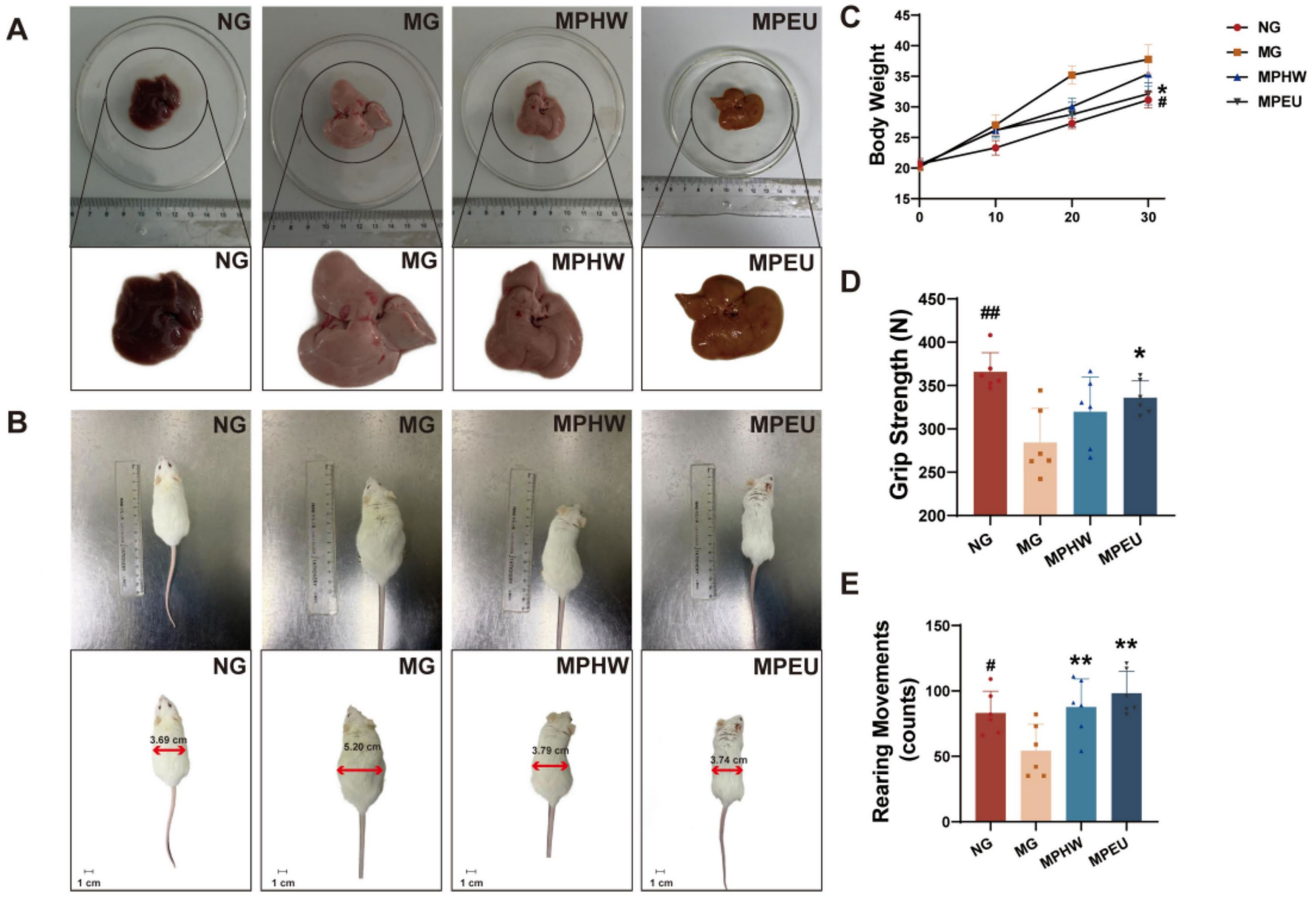
Effect of MP on overall condition of NAFLD (*n* = 6). **(A)** Liver morphology. **(B)** Images of mouse body morphology. **(C)** Changes in body weight. **(D)** Grip strength. **(E)** Rearing movements. Compared with the NG, ^#^*p* < 0.05, ^##^*p* < 0.01; Compared with the MG, ^*^p < 0.05, ^**^p < 0.01.

Liver tissue in the NG exhibited a reddish-brown color, soft texture, smooth surface, and sharp edges. In the MG, massive lipid accumulation within hepatocytes caused liver enlargement with a pale red appearance. Both the surface and cross-section displayed greasy appearance, brittle texture, and slight roughness. The MPEU and MPHW groups showed improvement compared to the model group (Figure 5A).

Mice with high-sugar, high-fat diet-induced fatty liver exhibited pronounced obesity phenotypes. This dietary pattern caused energy intake to far exceed expenditure, leading to excess energy deposition as fat in the liver and adipose tissues throughout the body. This process not only triggered hepatic lipid accumulation forming fatty liver but also significantly increased body weight and body fat percentage, manifesting obesity characteristics. Mice in the model group exhibited larger body size and significantly higher body weight than the normal group (*p* < 0.05), while mice in MPEU and MPHW groups showed reduced body size and significantly lower body weight (*p* < 0.05, Figures 5B,C).

High-sugar, high-fat diet-induced fatty liver triggers hepatic lipid accumulation, systemic oxidative stress, and inflammatory responses, while also inducing insulin resistance (39). This impairs skeletal muscle energy uptake and utilization, leading to reduced muscle strength manifested as decreased grip force. It further disrupts motor control in the central nervous system. Concurrent obesity increases exercise burden, consequently reducing the frequency of spontaneous activity in mice. Grip strength and spontaneous activity frequency were significantly reduced in the model group (*p* < 0.05 or 0.01), while MPEU and MPHW administration resulted in marked improvement (*p* < 0.05 or 0.01, Figures 5D,E).

### MP improves blood biochemical indicators in NAFLD mice

**Figure 6:**
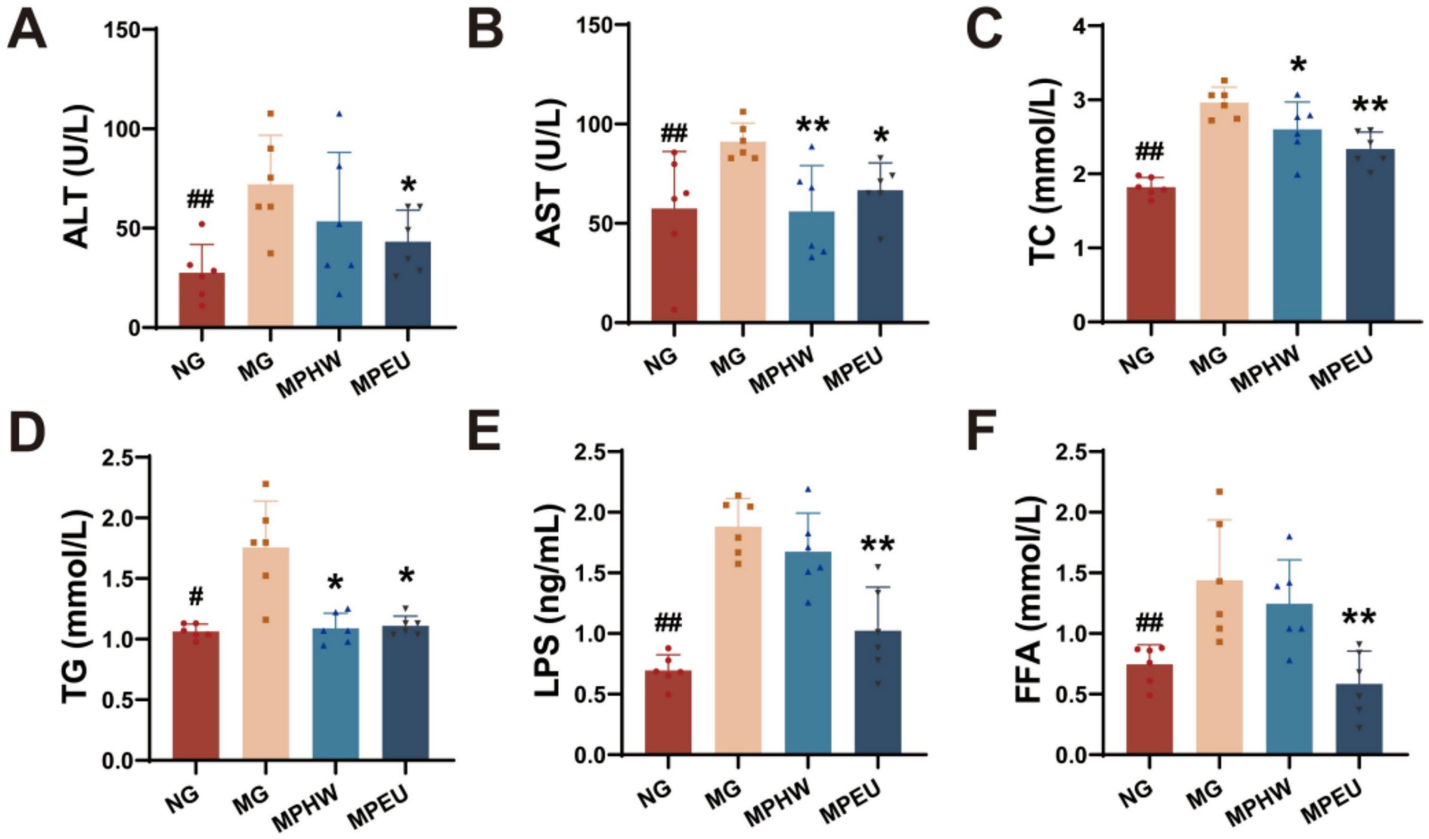
Effect of MP on blood biochemical indicators of NAFLD (*n* = 6). **(A)** ALT. **(B)** AST. **(C)** TC. **(D)** TG. **(E)** LPS. **(F)** FFA. Compared with the NG, ^#^*p* < 0.05, ^##^p < 0.01; Compared with the MG, ^*^p < 0.05, ^**^p < 0.01.

In the MG, ALT and AST, core markers of hepatocyte injury, were significantly elevated. This indicates that the high-sugar, high-fat diet caused excessive lipid deposition in hepatocytes, leading to cellular damage, increased cell membrane permeability, and massive release of intracellular transaminases into the bloodstream. TC and TG, key indicators of lipid metabolism, were significantly elevated, reflecting an imbalance in hepatic lipid synthesis and metabolic clearance. This led to substantial lipid accumulation and release into the bloodstream, resulting in lipid metabolism disorders and hyperlipidemia. In the MPEU group, ALT, AST, TC and TG levels all decreased significantly (*p* < 0.05 or 0.01, Figures 6A–D).

Lipopolysaccharide is a canonical biomarker of impaired intestinal barrier function and endotoxemia. Remarkably elevated LPS levels were detected in MG, which proved that the high-fat and high-fructose diet destroyed intestinal mucosal integrity and increased intestinal permeability. Such intestinal leakage allows LPS secreted by intestinal gram-negative bacteria to translocate into peripheral blood, further triggering hepatic inflammatory damage through the gut-liver axis. FFAs vital mediators of lipotoxicity, also accumulated drastically in MG mice. Excess FFAs transported to the liver induced severe lipotoxic stress and accelerated lipid deposition in hepatocytes. Notably, intestinal barrier injury induced by high-energy diet simultaneously elevated circulating LPS and FFAs, and the two substances synergistically worsened NAFLD. Notably, both LPS and FFA concentrations were significantly reduced after MPEU intervention (*p* < 0.01, Figures 6E,F).

### MP ameliorates liver injury in NAFLD mice

**Figure 7:**
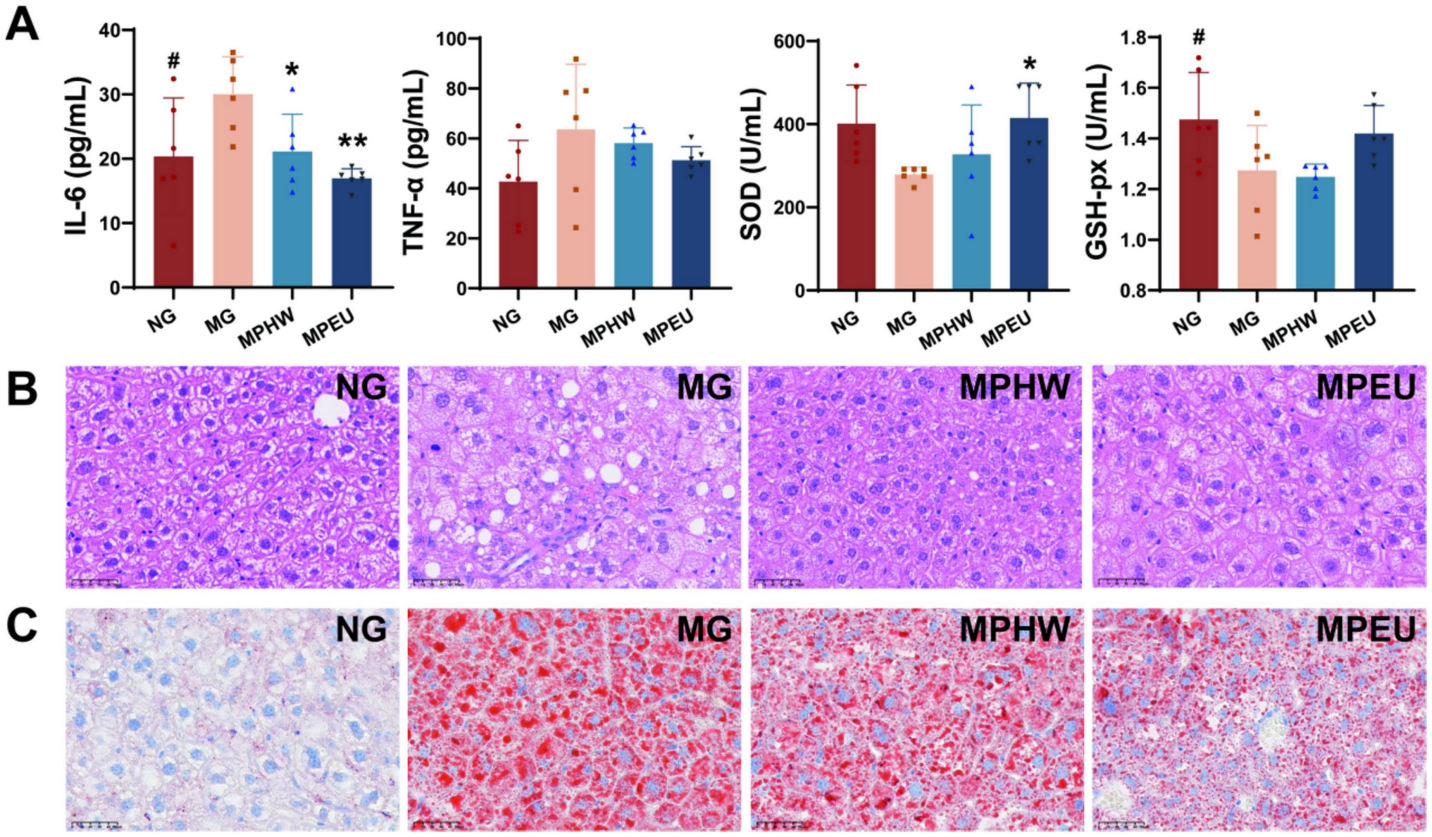
Improvement effect of MP on the liver of mice with NAFLD (*n* = 6). **(A)** Contents of IL-6, TNF-*α*, SOD, and GSH-px in liver homogenate. **(B)** HE staining. **(C)** Oil Red O staining. Compared with the NG, ^#^*p* < 0.05, ^##^*p* < 0.01; Compared with the MG, ^*^p < 0.05, ^**^p < 0.01.

In the MG, the content of IL-6 in liver homogenates was significantly elevated (*p* < 0.05), while TNF-*α* showed an upward trend. GSH-Px activity was significantly reduced (*p* < 0.05), and antioxidant enzyme SOD showed a decreasing trend, indicating that high-sugar, high-fat diet-induced fatty liver is accompanied by severe inflammatory response and oxidative stress damage in the liver. Following MPEU and MPHW intervention, IL-6 levels in liver tissue were significantly downregulated (*p* < 0.01), while TNF-α levels showed a decreasing trend. SOD activity significantly rebounded in MPEU (*p* < 0.05), and GSH-px activity exhibited a recovery trend (Figure 7A).

Hepatic tissue HE staining revealed that in the NG, hepatic lobule structures were intact and clearly defined, with hepatocytes arranged in an orderly fashion and cytoplasm uniformly devoid of lipid vacuoles. In contrast, the MG exhibited disrupted hepatic lobule architecture, with hepatocytes showing swelling and deformation. Cytoplasmic lipid vacuoles of varying sizes were abundant, and some hepatocytes displayed ballooning degeneration, indicating severe lipid accumulation and structural damage. Following MPEU and MPHW intervention, hepatic lobule architecture in mice tended toward restoration. The number and volume of hepatocyte lipid vacuoles significantly decreased, with cellular morphology largely returning to normal (Figure 7B).

Oil Red O staining results showed that only a small number of red lipid droplets were present in the liver tissues of mice in the NG. In contrast, liver tissue in MG exhibited extensive diffuse red lipid droplets, indicating massive hepatic lipid accumulation. MPEU and MPHW intervention markedly reduced the number and density of red lipid droplets in liver tissue, significantly improving hepatic lipid deposition (Figure 7C).

### MP alleviates intestinal injury and promotes repair

**Figure 8:**
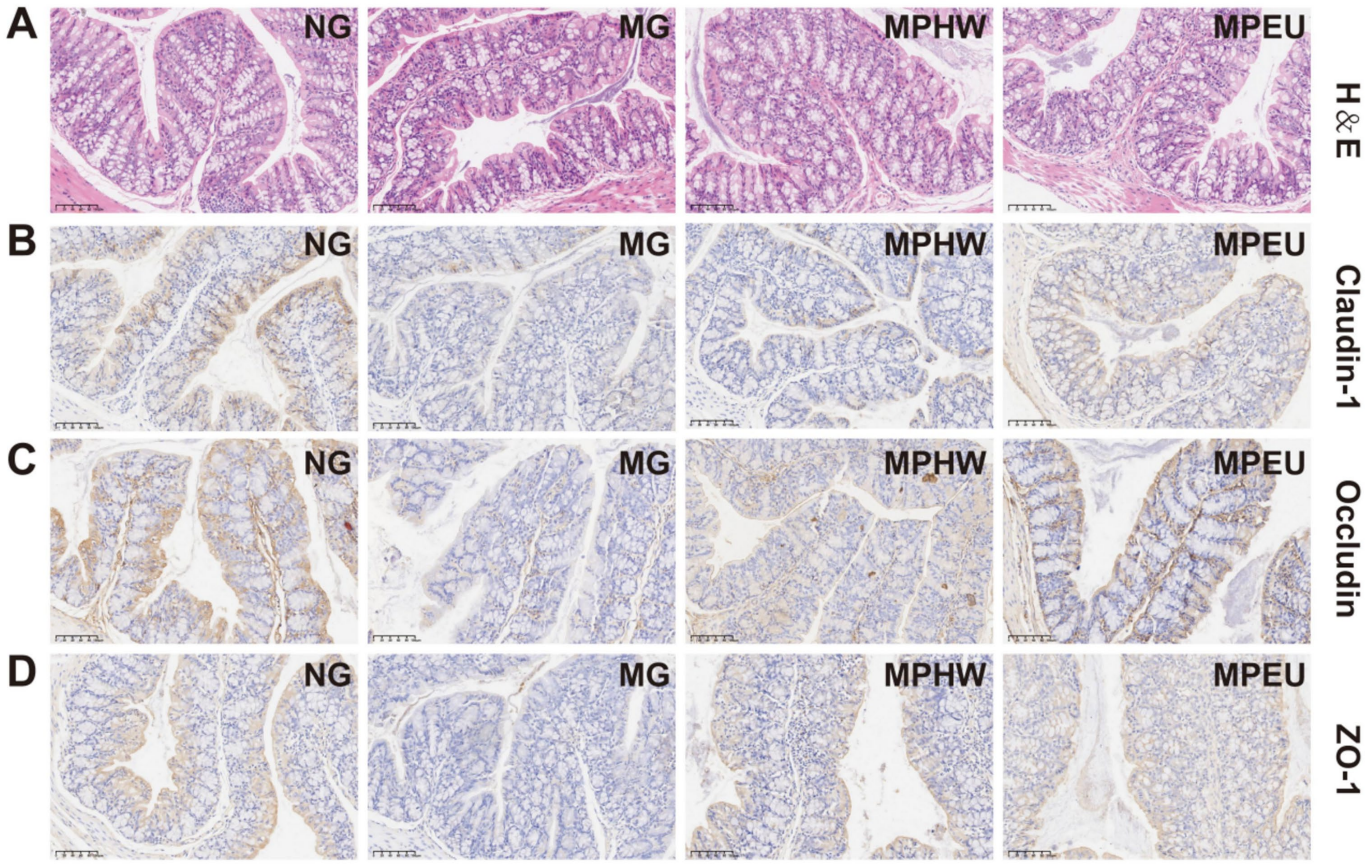
Effect of MP on the colon. **(A)** HE staining. **(B–D)** Colon IHC.

HE staining of colon tissue revealed intact mucosal architecture, well-organized epithelial cells, and regular villus height and crypt depth in the NG with no significant inflammatory infiltration. In contrast, the MG exhibited disrupted mucosal structure, damaged epithelial cells, obvious decrease in villus height and increase in crypt depth, and marked inflammatory cell infiltration. Following MP intervention, the colonic mucosal structure in both the MPEU and MPHW groups tended toward integrity, with well-organized epithelial arrangement, restored villus height and crypt depth, and significantly reduced inflammatory infiltration, with more pronounced improvement in the MPEU (Figure 8A).

Immunohistochemical results revealed strong brownish-yellow positive expression of tight junction proteins Claudin-1, Occludin, and ZO-1 between colonic epithelial cells in the NG. In the MG, the intensity of positive expression for these proteins was significantly reduced, indicating impaired intestinal tight junctions and decreased intestinal barrier function. Following MPEU and MPHW intervention, positive expression of tight junction proteins markedly increased across all groups, with superior repair observed in MPEU (Figures 8B–D).

The severe destruction of colonic tight junctions in the MG provides a direct structural basis for the elevated serum LPS described earlier. Broken intestinal barrier permeability triggers massive translocation of gut bacterial LPS into peripheral blood, while disordered intestinal lipid metabolism further promotes abnormal accumulation of circulating FFAs. Translocated LPS and excess FFAs jointly mediate inflammatory and lipotoxic injury to the liver via the gut-liver axis, which explains the core pathological link between intestinal barrier dysfunction and NAFLD progression. By restoring colon mucosal integrity and upregulating tight junction proteins, MPEU effectively inhibits LPS leakage and relieves FFA overaccumulation, cutting off the pathological crosstalk between gut and liver.

### MP regulation of gut microbiota and its metabolites

**Figure 9:**
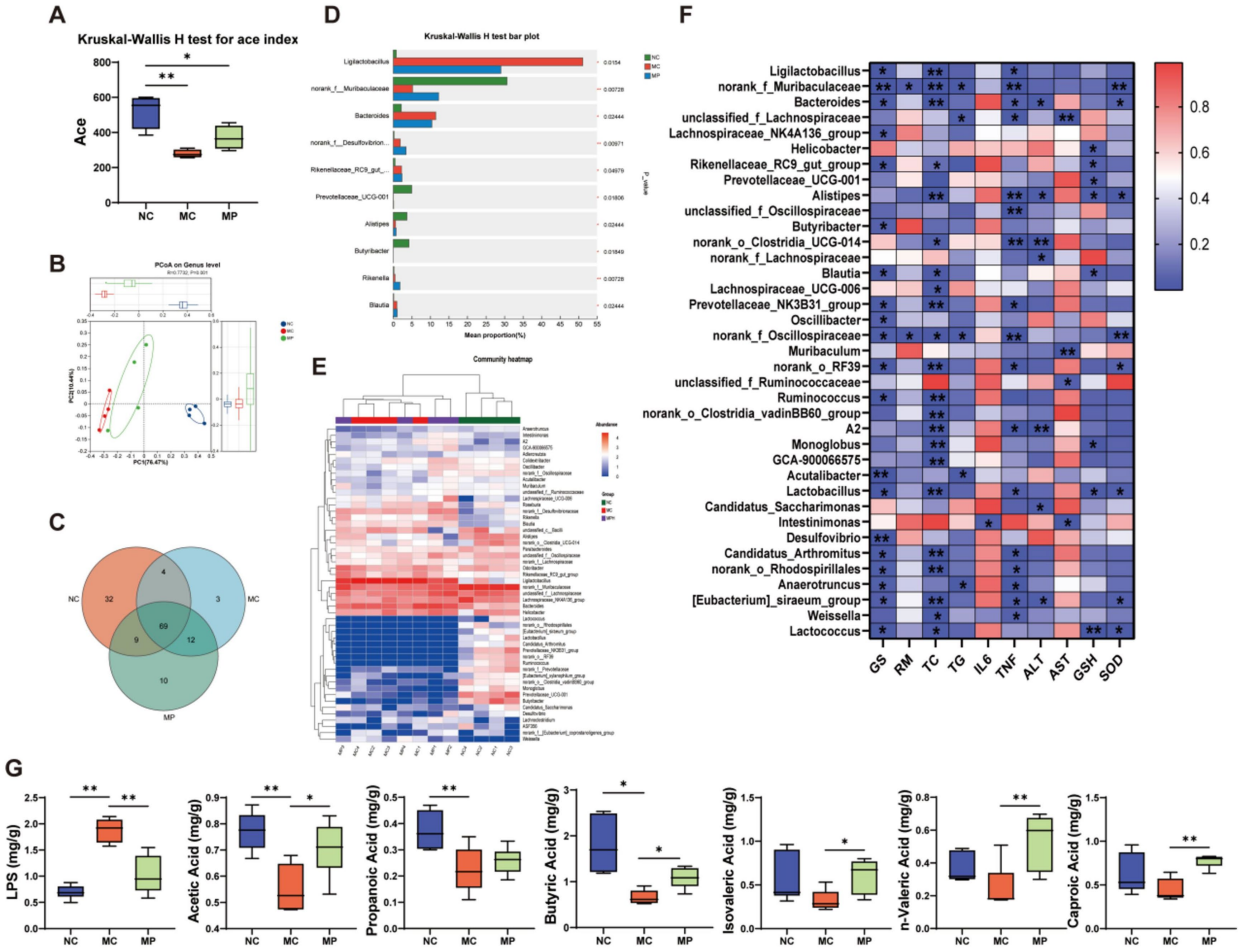
Effect of MP on intestinal flora and its metabolites in NAFLD mice (*n* = 4). **(A)** Alpha diversity. **(B)** PCoA clustering plot. **(C)** Venn diagram. **(D)** Statistics of differential flora among groups. **(E)** Community heatmap. **(F)** Clinical correlation analysis plot. **(G)** Metabolite content. ^*^p < 0.05, ^**^p < 0.01.

We sequenced the gut microbiota of animals across all groups. Results revealed significant differences in *α*-diversity between the NG and MG, with the MPEU group showing recovery relative to the MG (Figures 9A,C). PCOA analysis indicated distinct microbial compositions among groups (Figure 9B). These findings demonstrate that the microbiota composition of alcoholic fatty liver mice fed a high-fat diet differs from that of NG, while MPEU treatment improved dysbiosis in the MG (Figure 8B).

A heatmap displays the relative abundance of the top 50 bacterial genera. Analysis of the top 10 species by abundance revealed increased abundance at the genus level in NAFLD mice for: *Bacteroides*, *norank_f__Desulfovibrionaceae*, *Rikenellaceae_RC9_gut_group*, *Rikenella*, and *Blautia* increased, while *norank_f__Muribaculaceae*, *Prevotellaceae_UCG-001*, *Alistipes*, and *Butyribacter* decreased, indicating differences in microbial abundance among groups (Figures 9D,E).

Heatmaps revealed associations between distinct bacterial communities and pharmacological indicators. TC and TNF-α showed significant correlations with most bacterial genera, suggesting MP may improve NAFLD symptoms in mice by modulating the abundance of these microbial populations (Figure 9F).

Finally, we measured LPS levels in mouse blood and SCFA levels in feces. Elevated serum LPS levels in NAFLD model mice resulted from the combined effects of gut microbiota dysbiosis, intestinal barrier damage, and endotoxin translocation into the bloodstream. This finding, coupled with altered intestinal histopathology and intestinal barrier proteins, indicates severe intestinal barrier impairment. This barrier dysfunction facilitates the translocation of harmful substances into the bloodstream, exacerbating liver damage. Short-chain fatty acid results showed significantly reduced acetate and butyrate levels in the feces of NAFLD model mice (*p* < 0.05 or 0.01), while MP group mice exhibited significantly increased acetate and butyrate levels in feces (*p* < 0.05) (Figure 9G). Both core short-chain fatty acids produced by gut microbiota fermentation of dietary fiber and key metabolic products in host-microbiome interactions.

These findings suggest that MPEU’s therapeutic effect on NAFLD centers on the gut microbiota-intestinal barrier-liver lesion axis, exerting its efficacy through multi-target synergistic regulation. It significantly reduces the abundance of pro-inflammatory dysbiotic bacteria such as *norank_f_Desulfovibrionaceae* in the model, indirectly regulating gut microbiota homeostasis while promoting the production of short-chain fatty acids like acetate and butyrate. These short-chain fatty acids enhance intestinal barrier function, reduce intestinal permeability, block LPS entry into the bloodstream and subsequent activation of inflammatory pathways, thereby alleviating chronic liver inflammation and lipid metabolism abnormalities.

## Discussion

This study focused on optimizing the extraction process of MPEU and its ameliorative effects on NAFLD. Key findings are as follows: The extraction process was optimized using a Box–Behnken design combined with RSM and GA-BP neural networks. The RSM-predicted extraction rate 9.96% showed excellent agreement with the actual validated value (10.06%), whereas the GA-BP prediction (12.23%) exhibited significant deviation from the actual value (10.04%). Gut microbiota analysis revealed that MPEU intervention significantly regulated dysbiosis in the NAFLD model group, reduced the abundance of pro-inflammatory bacteria, and influenced SCFA production. Serum marker detection indicated that MPEU lowered LPS levels in model serum, thereby alleviating hepatic inflammation and oxidative stress, ultimately improving NAFLD pathology. Based on the above findings, an in-depth discussion on the extraction process of MPEU and its mechanism for improving NAFLD is presented below.

### Methodological considerations for process optimization: applicability of RSM and limitations of GA-BP

In the optimization of MPEU extraction processes, significant discrepancies exist between the predicted values from the GA-BP neural network and the actual values, whereas the deviation between RSM predictions and actual values is minimal. This disparity does not stem from inherent flaws in the GA-BP method itself, but rather arises from a combination of model adaptability, sample size limitations, and process characteristics (40). First, the core strength of GA-BP neural networks lies in handling complex systems with strong nonlinear interactions among multiple factors. However, their predictive accuracy is highly dependent on the size of the training sample and the completeness of its distribution (41). In this study, GA-BP modeling utilized only 29 sets of BBD experimental data, representing a typical small-sample modeling scenario. Moreover, the highest actual extraction rate in the training set was merely 10.54%, lacking high-value samples exceeding 11% as calibration references. When predicting 12.23%, a value beyond the training set range, GA-BP could only generate results through extrapolation, inevitably amplifying deviations. Second, the model exhibits mild overfitting. During training, GA-BP overlearned local nonlinear features in the training set, reducing its adaptability to unseen optimal process points. Simultaneously, the genetic algorithm’s optimization targeting maximized predicted extraction rate tends to favor parameter combinations yielding high predictions but low actual feasibility, further widening the gap between predicted and actual values (34).

Furthermore, in the MPEU extraction process studied here, interactions among influencing factors primarily exhibit moderate to low nonlinearity, eliminating the need for complex high-order nonlinear mapping models. The quadratic polynomial employed in RSM accurately captures the intrinsic relationship between process parameters and extraction rates. Its local fitting characteristics align well with the complexity of this process, resulting in superior prediction reliability. This highlights that process optimization methods should be tailored to the inherent nonlinear characteristics of the process rather than blindly pursuing complex models.

The GA-BP model was trained only on the 29 experimental runs generated by RSM central composite design, rather than adopting an independent expanded sample dataset for neural network training. Neural networks generally require a larger number of training samples to exert their nonlinear fitting advantages; the insufficient training data set weakens the predictive performance of GA-BP, resulting in unequal comparison conditions between the two algorithms. Therefore, the conclusion that “RSM shows better optimization performance than GA-BP” is only applicable to the limited sample size adopted in this study and cannot be generalized to all polysaccharide extraction process optimization systems. Subsequent comparative research should match sufficient independent training samples for the GA-BP model to achieve a fairer algorithm performance evaluation.

### Influence of extraction methods on polysaccharide structural features

The extraction method directly determines the physicochemical structure of polysaccharides, and structural integrity is the basis for their biological activities. In this study, we compared polysaccharides prepared by conventional MPHW and by MPEU, revealing notable differences at multiple structural levels.

First, in terms of extraction efficiency, the yield of MPEU (10.06%) was significantly higher than that of MPHW (7.06%), attributable to the synergistic effects of cellulase-mediated cell wall degradation and ultrasound-induced cavitation that promote polysaccharide dissolution. More importantly, structural characterization revealed profound impacts of extraction methods on molecular integrity. FT-IR spectra showed that the transmission-mode FTIR absorption valleys of MPEU at 3384.36, 2931.77, 1635.52 and 1031.58 cm^−1^ successively correspond to O-H, C-H, C=O and pyranose glycosidic bond vibrations, and the absence of the 1750 cm^−1^ esterified carboxyl signal indicates its low methylation degree despite containing galacturonic acid. Monosaccharide composition analysis revealed that MPEU consisted of mannose, galacturonic acid, glucose, galactose, arabinose, and fucose in a molar ratio of 48.64: 19.90: 13.78: 1.10: 0.69: 15.89; this rich compositional diversity provides a structural basis for its multi-target regulatory activities. SEM imaging showed that MPEU appeared as irregular granular aggregates with smooth, amorphous surfaces, without the fragmentation or porosity that would indicate thermal degradation, suggesting that the mild conditions of EAUE effectively preserved the native morphology of the polysaccharides.

Notably, the Congo red test indicated that MPEU lacks a triple-helix conformation, ruling out this structural feature as a contributor to its bioactivity. This finding suggests that the biological effects of MPEU are primarily derived from other structural factors, such as monosaccharide composition, glycosidic linkage types, and molecular weight distribution. Taken together, EAUE not only outperforms hot water extraction in yield, but more importantly, it offers a significant advantage in preserving structural integrity, which provides a structural rationale for the subsequent differences in biological activities.

### MPEU ameliorates NAFLD via the “gut microbiota-intestinal barrier-liver” axis–mechanistic insights

The core mechanism of MPEU’s improvement on NAFLD involves multi-level, multi-target synergistic intervention by regulating the gut microbiota-intestinal barrier-liver inflammation/oxidation axis. This mechanism aligns closely with the core pathophysiological feature of NAFLD: disruption of the gut-liver axis. As the initial segment of this axis, gut microbiota structural homeostasis directly influences intestinal barrier function and liver health (42). In this study, the NAFLD model group exhibited marked dysbiosis, characterized by significantly elevated abundances of pro-inflammatory Gram-negative bacteria such as *norank_f_Desulfovibrionaceae*, alongside substantial reductions in healthy gut core commensals like *norank_f_Muribaculaceae* and *Prevotellaceae_UCG-001*, among other core commensals of healthy intestines. This dysbiosis not only disrupted the gut microbiome balance but also laid the groundwork for subsequent intestinal barrier damage and hepatic inflammation (43–45). Following MPEU intervention, the abundance of disrupted bacteria such as *Ligilactobacillus*, *norank_f_Desulfovibrionaceae*, and *Bacteroides* was significantly reduced in the MG. Although the abundance of characteristic dominant bacteria in the healthy control group (NG group) was not fully restored, the intervention effectively suppressed further imbalance in the microbial community structure, providing a microbiological foundation for intestinal barrier repair and alleviation of liver damage.

The intestinal barrier serves as a critical link between gut microbiota and the liver, and its compromised integrity represents a pivotal point in the development of NAFLD (46). In this study, reduced abundance of healthy commensal bacteria in the model group led to decreased secretion of SCFAs such as acetate and butyrate. As the primary energy source for intestinal epithelial cells, the deficiency of butyrate directly impairs the proliferation and repair capacity of these cells, reduces the expression of tight junction proteins, and increases intestinal permeability (47). This allows LPS released by gram-negative bacteria undergoing lysis to pass through the damaged intestinal epithelium into the bloodstream, leading to elevated serum LPS levels (48). MPEU intervention modulates gut microbiota metabolism to promote SCFAs production, including acetate and butyrate. Notably, butyrate significantly enhances tight junction function and reduces intestinal permeability, thereby blocking LPS entry into the bloodstream. This mechanism aligns closely with the observed elevation of serum LPS in the model group and its subsequent reduction following MP intervention.

Lipopolysaccharide entering the bloodstream serves as a key trigger for liver inflammation and oxidative stress, acting as a central mediator in the gut microbiota-intestinal barrier-liver inflammation/oxidation axis (49). Elevated serum LPS enters the liver via the portal vein, binds to Toll-like receptor 4 (TLR4) on hepatocyte surfaces, activates the downstream NF-κB inflammatory signaling pathway, and induces massive release of pro-inflammatory factors such as TNF-*α* and IL-6, triggering chronic low-grade liver inflammation (50). Concurrently, persistent inflammatory activation boosts reactive oxygen species (ROS) generation and inhibits the activities of antioxidant enzymes (SOD, GSH-Px and others), thereby triggering hepatic oxidative stress imbalance (35). This exacerbates hepatocyte damage, lipid peroxidation, and fat accumulation, forming a vicious cycle: microbiome dysbiosis–intestinal barrier damage - LPS entry into the bloodstream - liver inflammation/oxidation–worsening NAFLD. MP effectively reduces LPS translocation by regulating gut microbiota and repairing the intestinal barrier. By reducing ROS production and enhancing antioxidant enzyme activity, MP breaks the vicious cycle, ultimately decreasing hepatic fat accumulation and hepatocyte damage to improve NAFLD. Furthermore, correlation analysis between gut microbiota and clinical markers confirmed significant associations between MP-regulated dominant bacteria and inflammation, oxidative stress, and metabolic indicators. This further substantiates the pivotal role of the “gut microbiota-intestinal barrier-liver inflammation/oxidation” axis in MPEU’s NAFLD improvement.

### Limitations of the study

Several limitations of the current research should be acknowledged. First, only four biological replicates per group were subjected to 16S rRNA high-throughput sequencing for gut microbiota profiling. Such a small sample size inevitably reduces statistical power and impairs the robustness of differential bacterial identification, which may obscure subtle but biologically relevant microbial alterations between groups. Second, the mechanistic exploration mainly relied on phenotypic detection of animal models and polysaccharide structural characterization, while *in vitro* cell-based validation experiments were relatively insufficient; the regulatory pathways of MPEU on intestinal epithelial barrier and inflammatory response require further cellular verification. Third, this study did not construct gene knockout or overexpression animal models to precisely confirm the core targets mediating the protective effect of MPEU, which restricts the reliability of the proposed signaling axis. Fourth, we only performed 16S rRNA sequencing to characterize gut bacteria without combining untargeted metabolomics to systematically clarify the crosstalk between gut flora-derived metabolites and host physiological indicators. Finally, all animal experiments were conducted on male mice alone, and sex-related differences in the intervention effect of MPEU remain unclear.

In future work, we will increase the number of sequencing samples to improve statistical reliability, supplement in vitro cell functional assays and gene-modified animal models, integrate multi-omics analysis, and compare the intervention effects in both male and female rodents to deepen and validate the underlying molecular mechanism of MPEU against intestinal dysfunction.

## Conclusion

In this study, RSM proved more reliable than GA-BP for optimizing the enzyme-ultrasound assisted extraction of mulberry polysaccharides. The optimal extraction process as follows: extraction time: 107 min; liquid-to-solid ratio: 33:1; enzyme addition amount: 350 mg; extraction temperature: 52 °C, achieving a yield of 10.06%. MPEU preserved structural integrity better than MPHW and exhibited superior efficacy in alleviating NAFLD in mice. Mechanistically, MPEU reshaped gut microbiota, reinforced intestinal barrier function, and reduced hepatic inflammation. These findings support EAUE coupled with RSM as a promising approach for producing high-quality mulberry polysaccharides as functional ingredients for NAFLD intervention.

## Data Availability

All sequencing data generated in this study have been deposited in the National Center for Biotechnology Information (NCBI) repository with BioProject accession number PRJNA1502788.
